# Exploring the Genetic Heritage of the Yucatán Black Hairless Pig: A Comparative Worldwide ROH Study

**DOI:** 10.3390/vetsci13010054

**Published:** 2026-01-07

**Authors:** Jorge Barzilai Lara-Castillo, Clemente Lemus-Flores, Raúl Sansor-Nah, Néstor Gerardo Michel-Regalado, Fernando Grageola-Núñez, William Orlando Burgos-Paz, Job Oswaldo Bugarín-Prado

**Affiliations:** 1 Posgrado en Ciencias Biológico Agropecuarias, Universidad Autónoma de Nayarit, Tepic 63315, Mexico; 22000023@uan.edu.mx (J.B.L.-C.); nestor.michel@academicos.udg.mx (N.G.M.-R.); 2 Unidad Académica de Medicina Veterinaria y Zootecnia, Unidad Académica de Agricultura, Universidad Autónoma de Nayarit, Tepic 63315, Mexico; clemus@uan.edu.mx (C.L.-F.); fgrageola@uan.edu.mx (F.G.-N.); 3 Asociación Mexicana de Criadores de Cerdos de Origen Ibérico Yucatán, Tizimín 97705, Mexico; raul-sannah@hotmail.com; 4 Corporación Colombiana de Investigación Agropecuaria—Agrosavia, Centro de Investigación Turipaná, Km 13 Vía Montería, Cereté, Córdoba 250047, Colombia; wburgos@agrosavia.co

**Keywords:** runs of homozygosity, Yucatán Black Hairless Pig, indigenous breeds, GGP porcine 50K, ROH, Mexican breed

## Abstract

The Yucatán Black Hairless Pig (YBHP) is an indigenous Mexican breed shaped by tropical environments and traditional management systems. This study aimed to characterize its runs of homozygosity (ROH) and compare its ROH patterns with those of indigenous and commercial pig breeds worldwide using the GGP Porcine 50K SNP array. After applying standard quality-control filters, ROH were identified, classified by length, and evaluated for shared homozygous regions across populations. The YBHP showed intermediate levels of genomic homozygosity (FROH = 0.09), with most ROH segments falling within the 5–20 Mb range. Comparative analyses indicated that the YBHP shared a higher number of ROH segments with indigenous populations than with cosmopolitan breeds. Gene annotation within ROH regions revealed SNPs located in genes previously reported in indigenous populations, including FGF5, BMP2K, PAQR3, RASGEF1B and ANTXR2, which participate in developmental and regulatory biological pathways. Overall, these results provide a detailed description of ROH distribution in the YBHP and offer complementary information to previous studies on its genetic characterization, supporting future conservation and management strategies.

## Simple Summary

The Yucatán Black Hairless Pig is an indigenous Mexican breed adapted to tropical conditions. This study characterized its homozygosity using genome-wide SNP data and compared its runs of homozygosity (ROH) with those of indigenous and commercial pig populations worldwide. The results indicate moderate genomic inbreeding and the presence of conserved homozygous regions shared mainly with other indigenous breeds. These findings provide complementary genomic information useful for the characterization and conservation of this local breed. Protecting this genetic heritage is important for biodiversity conservation and for promoting sustainable livestock production in tropical regions of México.

## 1. Introduction

The domestication of Pigs (Sus scrofa) represents one of the most significant milestones in the co-evolutionary events in history between humans and animals, leading to a vast genetic diversification shaped by both natural and artificial selection [1]. Over centuries, swine breeds have adapted to distinct ecological niches and productive systems, resulting in a wide spectrum of morphological, physiological, and behavioral characteristics [2,3]. The continuous improvement in genomic and rapid development of genomic technologies, especially high-density SNP genotyping and whole-genome resequencing, has enabled researchers to unravel the genetic architecture underlying these processes, reconstructing demographic histories, admixture patterns, and selection footprints across both commercial and indigenous pig breeds [4,5].

Among the most informative genomic tools, Runs of Homozygosity (ROH), continuous segments where alleles are identical by descent, have proven to be powerful indicators of population history, inbreeding, and selection [4,6]. ROH arise through demographic bottlenecks, genetic drift, or recent inbreeding, and their length distribution provides insight into the temporal scale of these events: long ROH suggest recent common ancestry, while shorter ones reflect more ancient, shared origins [7,8]. In livestock, this approach has become essential for quantifying genomic autozygosity (FROH) and identifying regions enriched in homozygosity that may harbor genes influencing adaptation, reproduction, or production traits [3,9].

Recent advances in computational genomics and high-throughput genotyping platforms such as the GeneSeek GGP 50K/80K arrays have standardized ROH-based analyses across multiple species, allowing reliable detection of selection signatures and genomic inbreeding patterns in domestic and wild animals. Studies in breeds such as the Large White, Ding’an, and Banna miniature pigs have demonstrated that ROH landscapes mirror historical selection pressures and can reveal genomic regions associated with fertility, muscle development, fat deposition, and disease resistance [10,11]. These findings emphasize the role of ROH as integrative indicators linking population structure, demographic history, and adaptive traits.

Beyond production-oriented breeds, local pig populations constitute invaluable reservoirs of genetic diversity shaped by centuries of environmental adaptation and traditional husbandry. Their genomes often retain polymorphisms conferring rusticity, environmental resilience, and superior meat quality [12,13].

Within this context, the Yucatán Black Hairless Pig (YBHP) represents a remarkable example of genetic conservation and adaptation to tropical environments. Previous research has reported that this local Mexican breed retains a distinctive ancestral background closely related to Iberian pigs, with limited introgression from cosmopolitan commercial lines [14,15]. This genetic and natural isolation, driven by the geographical remoteness of its environment and the selective pressures of a tropical climate together with traditional extensive management practices, has contributed to traits such as heat tolerance, rusticity, moderate growth, and a meat quality highly appreciated for its flavor and texture [16]. However, although previous studies have characterized the genetic diversity and population structure of the Yucatán Black Hairless Pig [14], the genomic features associated with its phenotypic and adaptive attributes remain insufficiently described. Information regarding the extent, distribution, and length classes of ROH segments in this breed is limited, and autozygosity has not yet been systematically assessed. In particular, there is scarce evidence on the presence of ROH islands potentially shaped by demographic processes or selection, and on how these patterns compare with those observed in other indigenous and commercial pig populations. Generating this information may contribute to a more precise description of homozygosity patterns in the breed, provide context regarding recent demographic tendencies, and support the identification of candidate genes located within conserved ROH segments.

Therefore, the present study aims to identify and characterize Runs of Homozygosity in the YBHP and compare them with those observed in indigenous and commercial pig populations worldwide. By detecting shared and exclusive ROH regions, this work seeks to describe differences in homozygosity patterns among populations and to identify genomic segments that may contain genes of potential biological relevance. This comparative approach provides complementary information to previous population-based analyses and contributes to a more detailed characterization of the genomic architecture of the breed.

## 2. Materials and Methods

### 2.1. Animals, Sampling, and Ethical Considerations

Due to the absence of an official population census and limited pedigree documentation, all accessible animals in the Yucatán Peninsula that exhibited the phenotypic characteristics of the YBHP were considered for inclusion. The population analyzed in this study corresponds to the same dataset previously described and characterized lines [14,15]. Briefly, animals were identified in the Yucatán Peninsula based on the traditional phenotypic criteria of the breed, and preference was given to individuals assumed to be unrelated to minimize redundancy. A total of 141 animals from different farms were included in the genomic dataset managed by the Mexican Association of Iberian-Origin Pig Breeders [17]. No additional biological sampling was performed for the present work, as this study is based exclusively on previously generated genotypes. All procedures related to the original data collection were conducted in accordance with national animal welfare regulations (NOM-062-ZOO-1999 and NOM-033-SAG/ZOO-2014) [18,19].

### 2.2. Genotyping and Dataset Integration

Genotyping was carried out by NEOGEN Corporation using the GeneSeek GGP Porcine 50K SNP array, which includes 50,967 markers distributed across all autosomes. This medium-density chip was selected due to its extensive validation in multiple pig breeds, cost-effectiveness, and reproducibility in population-genomic studies. Although this platform provides broad genome coverage, its design originally optimized for commercial breeds may limit the detection of rare or population-specific variants in underrepresented indigenous breeds such as the YBHP [10]. Nevertheless, extensive evidence supports its effectiveness in identifying homozygosity regions and selection signals, providing biologically meaningful information even within density constraints.

To enable comparative analyses, publicly available SNP genotype data from diverse indigenous and cosmopolitan pig populations worldwide were incorporated (see Table 1). These included local breeds from Africa, Asia, Europe, and the Americas, as well as commercial lines such as Duroc, Landrace, Large White, Pietrain, and Hampshire [20]. All external datasets were filtered and standardized following the methodological criteria described in their respective original publications [20]. Genomic coordinates were updated to the Sus scrofa 11.1 reference assembly, and only autosomal markers (chromosomes 1–18) were retained for downstream analyses.

### 2.3. Quality Control and Filtering

Quality control of the merged dataset was performed using PLINK v1.9 [21]. SNPs with a minor allele frequency (MAF) < 0.05 (--maf 0.05), a genotyping rate < 90% (--geno 0.1), or individuals with more than 10% missing genotypes (--mind 0.1) were excluded. Only SNPs with consistent autosomal mapping were retained. Breeds represented by fewer than five individuals were removed to prevent bias from underrepresented groups. After quality filtering, the final dataset comprised 506 individuals and 28,398 autosomal SNPs.

Individual heterozygosity values were inspected to identify potential outliers, and only high-quality genotypes were used for downstream analyses. The total autosomal genomic length covered by the SNP chip (L_AUTO) was calculated as the sum of physical ranges per chromosome and used in the computation of genomic inbreeding coefficients.

### 2.4. Detection of Runs of Homozygosity (ROH)

Runs of Homozygosity (ROH) were identified using PLINK v1.90 [21]. ROH were defined according to the following parameters: (i) a minimum of 50 SNPs per sliding window; (ii) one heterozygous genotype and no more than two missing SNPs were allowed per window; (iii) a minimum ROH length of 1 Mb to reduce linkage disequilibrium effects; (iv) a minimum SNP density of one SNP per 100 kb; and (v) a maximum gap of 500 kb between consecutive SNPs to prevent merging distant regions [10].

All parameters were selected to ensure comparability with recent porcine genomic studies and to preserve accuracy given the 50K SNP density of the array. Detected ROH were classified into five length categories (1–5, 5–10, 10–20, 20–40, and >40 Mb) for downstream descriptive analyses [22].

### 2.5. Descriptive Statistics and Genomic Inbreeding

Descriptive statistics of ROH per individual and population, including the average ROH length, average number of segments, and genomic inbreeding coefficient (FROH), were calculated in R v4.3.0 from PLINK output files [21,23]. The FROH value was computed as the proportion of the genome contained within ROH segments (FROH = LROH/LAUTO), providing a measure of genomic inbreeding independent of pedigree data. Breed-level summaries and frequency distributions of ROH segments by chromosome and length category were obtained using the packages dplyr, ggplot2, and data.table [23]. These descriptive analyses generated comparative statistics across the studied.

### 2.6. Detection of Common and Shared ROH Segments

To identify regions of high homozygosity shared among individuals, the frequency of occurrence of each SNP within ROH was calculated across individuals of each population. The top 1% of SNPs in the frequency spectrum were defined as ROH hotspots (or ROH islands), indicating genomic regions potentially under selection or influenced by shared ancestry [10]. For comparative purposes, ROH segments shared among populations were identified by intersecting genomic coordinates of ROH islands. Populations were grouped according to geographic origin: African, American, Asian, European, and Cosmopolitan. To illustrate the extent of overlap among populations, Venn diagrams were constructed using the eulerr package in R v4.3.0. [23] The analysis was based on ROH segments corresponding to the top 1% of SNPs with the highest frequency of occurrence within homozygous regions in each group. For each population comparison, only the ROH island segments shared with the YBHP were retained for subsequent analyses. The genomic length and all SNPs contained within these shared segments were extracted and used as the basis for downstream gene annotation and functional enrichment assays.

### 2.7. Pathway and Functional Analysis

Candidate genes were annotated via the Ensembl database (Sus scrofa 11.1, http://www.ensemble.org/; accessed on 30 October 2025) at 100 kb regions (upstream 50 kband downstream 50 kb) flanking the SNPs of ROH hotspots. Candidate genomic regions identified from ROH analyses were functionally annotated. Genomic coordinates corresponding to the shared and island ROH regions were intersected with annotated gene features from the GTF file using R (v4.3) [23] packages GenomicRanges and rtracklayer to extract all genes located within or overlapping these homozygous tracts. The resulting gene lists were then subjected to Gene Ontology (GO) and KEGG and GO Biological process pathway enrichment analyses using ShinyGO v.85.1 [24]. Overrepresented biological processes, molecular functions, and pathways were identified with a false discovery rate (FDR) < 0.05. The enriched categories provided insights into potential functional mechanisms and selective pressures acting on the YBHP and related populations. Functional clustering results were summarized graphically and tabulated for major categories relevant to metabolism, growth, adaptation, and stress response.

## 3. Results

### 3.1. Distribution of ROHs

A total of 8580 runs of homozygosity (ROH) were identified across all analyzed individuals. Table 2 shows the number of ROH fragments according to length class, ranging from 1 Mb to over 40 Mb. The average ROH length per fragment was 7.1 Mb, varying between 5.2 Mb and 8.42 Mb. ROH segments of 5–10 Mb represented the largest proportion, accounting for 52.9% (4543 ROH) of the total detected fragments. Segments between 10–20 Mb comprised 29.4% (2527 ROH), while short ROH of 1–5 Mb accounted for 8.4% (717 ROH). Longer segments of 20–40 Mb and >40 Mb were less frequent, contributing 8.3% (714 ROH) and 0.9% (79 ROH), respectively. Overall, most ROH fragments were concentrated within the 5–20 Mb range, with few segments exceeding 40 Mb.

**Figure 1:**
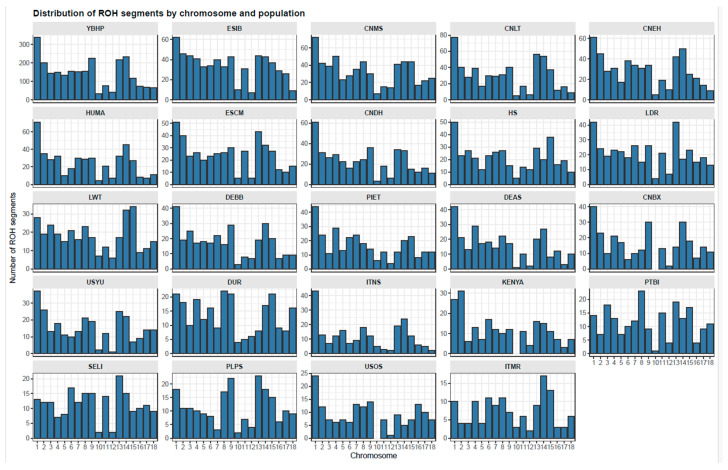
Distribution of ROH segments by chromosome and population. Variation in the number of ROH was observed across chromosomes. In most populations, the highest counts appeared on chromosome 1, forming a recurrent hotspot of homozygosity, whereas the lowest counts were consistently recorded on chromosome 10.

Figure 1 shows the distribution of ROH segments by chromosomes and population. Variation was observed among chromosomes and across populations, with certain regions displaying clear ROH concentration patterns. In most populations, the highest number of ROH segments occurred on chromosome 1, indicating a recurrent hotspot of homozygosity. Conversely, chromosome 10 consistently showed the lowest number of ROH across nearly all populations. Several breeds presented distinctive peaks in the first chromosomes, while others displayed a more uniform distribution across the genome.

**Figure 2:**
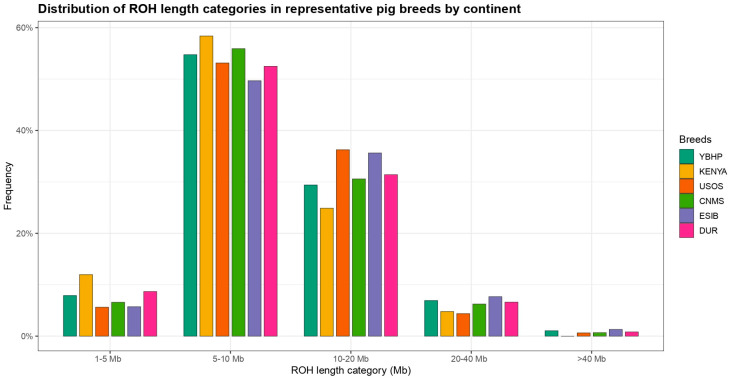
Frequency distribution of ROH length categories across representative populations. Segments in the 5–10 Mb class were the most frequent, followed by those of 10–20 Mb. Longer ROH (>40 Mb) were uncommon in all populations, and most fragments were concentrated within the 5–20 Mb range.

Figure 2 illustrates the distribution of ROH length categories in representative pig breeds from different continents. Across all groups, 5–10 Mb fragments were the most frequent, followed by 10–20 Mb. The categories 20–40 Mb and >40 Mb were comparatively rare in all populations. Overall, the majority of ROH segments were concentrated within the 5–20 Mb range, with minor variation between breeds, as shown.

The average number and length of ROH segments across all pig populations are summarized in Table 3. ROH were classified into five length categories (1–5 Mb, 5–10 Mb, 10–20 Mb, 20–40 Mb, and >40 Mb). Among all populations, the PIET breed exhibited the longest average ROH length (11.76 ± 9.06 Mb), whereas the KENYA and CNBX populations presented the shortest mean values (9.17 ± 4.98 Mb and 9.17 ± 4.37 Mb, respectively). The YBHP showed an intermediate mean ROH length of 10.64 ± 7.46 Mb. The mean number of ROH per individual varied considerably among populations. The highest value was observed in ESIB (30.60 ± 11.44), while the lowest occurred in DUR (12.74 ± 8.53). The YBHP population displayed a mean of 19.19 ± 14.27, placing it within the intermediate range. Regarding the inbreeding coefficient (FROH), the highest average was detected in ESIB (0.1543 ± 0.0676), followed by populations with moderate values such as PIET and CNBX, while DUR exhibited the lowest value (0.0596 ± 0.0476). The YBHP breed showed a moderate FROH of 0.0906 ± 0.0800, consistent with its intermediate average ROH length and count.

**Figure 3:**
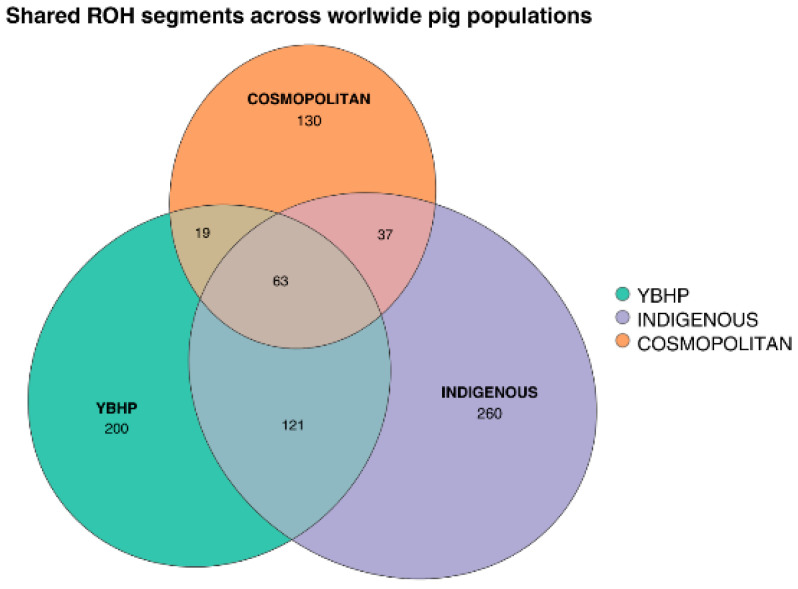
Venn diagram showing the overlap of ROH segments within the top 1% of shared homozygosity regions among the YBHP, indigenous, and cosmopolitan populations.

For comparative interpretation, breeds were grouped according to their general population category rather than strict geographic origin. Indigenous populations were considered as locally adapted breeds maintained outside intensive breeding schemes, whereas cosmopolitan breeds were defined as widely disseminated commercial lines developed under structured selection programs. These categories were used to summarize ROH patterns and to facilitate the visualization of shared and exclusive ROH segments across broad population types in the Venn diagram presented in Figure 3. This figure shows the overlap of ROH segments a total of 403 ROH were detected in YBHP, 481 in indigenous populations, and 249 in cosmopolitan breeds. The greatest overlap was observed between YBHP and indigenous populations, sharing 121 ROH, while 63 ROH were common to all three groups. The overlap between YBHP and cosmopolitan populations included 19 shared segments, and 37 segments were shared exclusively between cosmopolitan and indigenous groups, with no overlap from YBHP.

### 3.2. Candidate Genes

The 205 candidate genes identified within ROH in pigs were subjected to functional enrichment analyses (Table 4) [24]. Detailed enriched functional categories of ROH-associated genes for each continental group are provided in the Supplementary Materials (Tables S1–S5). Gene Ontology (GO) analysis revealed significant enrichment of specific biological processes with 95 genes. The comparative analysis among indigenous pig populations from Africa, America, Asia, and Europe identified a conserved set of five genes ANTXR2, BMP2K, FGF5, PAQR3, and RASGEF1B consistently present across all indigenous breeds. Two additional transcontinental gene-sharing patterns were observed among indigenous populations. GTF2H5 and SNX9 were common to breeds from America, Asia, and Europe, whereas SERAC1, SYNJ2, and SYTL3 were detected in African, American, and European breeds. The genes SERAC1, SYNJ2, and SYTL3. In European breeds, recurrent presence of LIF, PPARGC1B, GRB10, and CSF1R were identified. The cosmopolitan group exhibited a distinct set of exclusive genes ACP6, ANXA9, ARNT, BCL9, CDC42SE1, CTSS, GABPB2, HJV, ITGA10, MCL1, PDZK1, PI4KB, PIP5K1A, POLR3C, PSMD4, RFX5, SETDB1, SNX27, TXNIP, and VPS45 which were not detected in any indigenous population.

## 4. Discussion

A total of 8580 ROH were identified across all analyzed individuals, distributed in length classes ranging from 1 Mb to over 40 Mb. The mean ROH length was 7.1 Mb (5.2–8.4 Mb). Considering the medium density of the genotyping array (GeneSeek GGP Porcine 50K), these values represent moderate to short ROH in biological terms, since the limited SNP coverage restricts the detection of smaller haplotypes that would be captured by whole-genome sequencing approaches [9,10,25]. Consequently, the 5–10 Mb class, which accounted for 52.9% of all fragments, likely reflects ancestral homozygosity or historical inbreeding, rather than very recent parental relatedness. The distribution of ROH lengths was consistent with reports in other local or conserved breeds genotyped with similar platforms, such as Nero Lucano and Ding’an pigs, where most ROH ranged between 4 and 10 Mb and the mean ROH length remained below 10 Mb [9,25]. Shorter ROH (1–5 Mb) represented 8.4% of fragments, whereas 10–20 Mb tracts accounted for 29.4%, and segments > 20 Mb were rare (9.2% combined). In our dataset, this pattern is consistent with what is typical of moderately inbred populations subjected to selective pressures, showing neither the extensive long-range homozygosity characteristic of commercial breeds under intensive selection, nor the highly fragmented profile seen in ancient, diverse lineages [9,26].

Chromosome-specific variation was evident, with the highest ROH density observed on chr 1, a region previously identified as a recurrent hotspot of homozygosity in both indigenous and commercial pigs [10,26]. Conversely, chr 10 showed the lowest frequency of ROHs across populations. The accumulation of medium-sized tracts on the larger autosomes might reflect areas that have kept some level of LD or selection over time, although this cannot be stated with certainty. Overall, the predominance of 5–20 Mb fragments, together with the limited number of tracts exceeding 40 Mb, supports a demographic scenario of moderate inbreeding and some degree of selection, consistent with patterns observed in local breeds maintained under semi-conservation or low-intensity selection regimes [10,25]. The average ROH length varied between 9.17 Mb (KENYA, CNBX) and 11.76 Mb (PIET), with the YBHP presenting an intermediate mean of 10.64 ± 7.46 Mb. The number of ROHs per individual ranged from 12.74 in DUR to 30.60 in ESIB, while the genomic inbreeding coefficient (FROH) fluctuated between 0.0596 (DUR) and 0.1543 (ESIB), again with YBHP in an intermediate position (0.0906 ± 0.0800). These results indicate considerable variation in homozygosity among breeds, which likely relates to differences in their demographic backgrounds and how they have been managed. Similar numerical ranges have been reported in European local pig populations, where FROH values commonly span 0.08–0.15 and mean ROH lengths between 8 and 12 Mb are typical of traditional breeds maintained under low-intensity selection [27,28]. In contrast, commercial lines such as Large White and Pietrain generally display longer ROHs and higher FROH values due to artificial selection and closed breeding structures [10,26], whereas African and Asian indigenous pigs typically present lower inbreeding coefficients (FROH < 0.07) and shorter ROH, indicative of larger effective population sizes and historical gene flow [11,29].

A clear distinction emerges when comparing populations by geographic origin. European breeds, such as ESIB and PIET, exhibited higher levels of homozygosity, longer ROHs, and larger FROH values. Authors described comparable patterns in Mediterranean pigs, where medium to long ROHs (5–20 Mb) predominate as a legacy of sustained but controlled selection within small, structured populations [25,28].

Asian local breeds, including CNBX, displayed moderate values of both ROH number and FROH, consistent with results reported for Ding’an and Taihu Basin pigs, which showed intermediate genomic inbreeding (FROH 0.08–0.12) and ROH distributions concentrated in 4–10 Mb intervals [9,30]. This correspondence suggests that Asian indigenous breeds commonly retain balanced levels of homozygosity while maintaining broader genomic diversity, a pattern also noted in earlier studies of regional indigenous population.

African populations (KENYA) revealed the lowest FROH and shortest ROH, consistent with findings from Ugandan smallholder pigs, where low consanguinity (FROH ≈ 0.04) and scarce long tracts are attributed to extensive admixture and open mating systems [29]. These contrasts across continents confirm that ROH-based inbreeding is shaped by both demographic and selective histories, and that breeds maintained in traditional, non-industrial settings tend to conserve higher genomic diversity [11,12].

Within this comparative framework, the YBHP shows ROH metrics that fall within the central range of the studied populations. Its value does not indicate the elevated inbreeding levels typically observed in some cosmopolitan commercial lines. Nor do they correspond to the wider genomic dispersion often reported in highly diverse African or Asian populations. Rather, the YBHP displays a balanced ROH pattern that fits within the expected variability of locally adapted breeds, reflecting a conserved lineage without evidence of excessive inbreeding. Its mean ROH length (10.64 Mb) and FROH (0.09) are comparable to those reported for the Nero Lucano and Casertana pigs [25,28], indicating a similar degree of moderate genomic inbreeding. Within the American populations, it is also relevant to distinguish the indigenous YBHP from the USYUC. Although both share a geographical and historical link, the latter originates from animals exported from Mexico during the mid-20th century and has since been maintained as a closed laboratory population under controlled breeding programs [31]. Consequently, the USYUC represents a genetically differentiated lineage shaped by biomedical management rather than by traditional production or environmental adaptation, and its genomic configuration cannot be assumed to be equivalent to that of the native Mexican population [32]. The predominance of ROH in the 5–20 Mb interval, coupled with a moderate number of tracts per individual, suggests historical isolation and partial selection, likely reflecting both its Iberian origin and adaptation to tropical environments. This genomic profile supports the idea that YBHP experienced limited recent inbreeding, yet retained older autozygous regions formed through past demographic contractions and founder effects. As in other local breeds, these medium-length ROH may reflect the combined effects of small effective population size, genetic drift, and episodic selective pressures [9,25,28,30]. Despite the inherent limitations of medium-density SNP arrays, previous analyses using ancestry-informative markers have demonstrated clear Iberian ancestry in the YBHP [17]. The ROH patterns identified in this study are consistent with those earlier results, strengthening the interpretation that part of the autozygous genome reflects these ancestral lineages. Previous analyses based on ADMIXTURE reported that the YBHP retains a measurable proportion of Iberian ancestry [14,17], consistent with historical introductions during the colonial period. In this context, the concordance of ROH patterns between YBHP and Mediterranean or Southern European local breeds observed in our study supports the view that part of its genome still preserves ancestral autozygous tracts derived from those early lineages. These segments appear to have been maintained through continued reproduction within small, semi-isolated populations, rather than through recent admixture events. Therefore, occasional outcrossing events, together with recombination, may have disrupted extended homozygous tracts while preserving older conserved regions shaped by environmental adaptation and low-intensity selection. Comparative evidence across continents demonstrates that ROH patterns are consistent with breed histories and management intensity. The elevated homozygosity values observed in ESIB and PIET are consistent with the intense artificial selection and breeding consolidation historically reported for cosmopolitan and some European lines [28]; the lowest in KENYA and CNBX reflect open mating and historical admixture; and the YBHP occupies a balanced intermediate range, indicative of a semi-isolated, locally adapted population [2]. Moderate FROH values and the predominance of medium-sized ROHs have been reported as genomic signatures of breeds that retain variability despite demographic constraints [28]. The YBHP genome does not show clear signs of strong selective homogenization or of being heavily fragmented by genetic drift. Instead, it reflects the persistence of an ancestral genetic structure stabilized under adaptation to tropical environments, consistent with its known historical and zootechnical background [14,25,26].

A total of 205 genes were identified within regions of homozygosity (ROH) across the analyzed pig populations, 95 of which were significantly enriched in Gene Ontology (GO) biological processes. The results highlight functional patterns that distinguish indigenous from cosmopolitan breeds, which is consistent with their different selection histories. Across the indigenous populations, the genes ANTXR2, BMP2K, FGF5, PAQR3, and RASGEF1B appeared within shared ROH segments. Their repeated appearance probably reflects the presence of conserved haplotype blocks, something commonly seen in indigenous breeds [3]. Previous genome-wide scans in local European and Chinese pigs reported FGF5 and BMP2K as key regulators of morphogenesis, hair growth, and osteogenic differentiation under moderate selection pressures [10,27]. PAQR3 and RASGEF1B are components of insulin and Ras/Rap signaling pathways associated with growth regulation and cellular communication [9,26], whereas ANTXR2 contributes to tissue organization and extracellular matrix remodeling, functions relevant to structural adaptation and integumentary traits. The simultaneous detection of these genes in ROH segments from indigenous populations on different continents indicates that they recur within conserved homozygous regions across breeds. However, they do not originate from a single shared ROH; rather, they appear in different segments that commonly form part of stable haplotypic blocks in indigenous lineages.

Two additional transcontinental patterns were noted: GTF2H5 and SNX9 appeared in breeds from America, Asia, and Europe, while SERAC1, SYNJ2, and SYTL3 were found in African, American, and European pigs. Similar groups of genes have been reported in ROH regions of local European breeds, where they have been associated with pathways related to DNA repair and endocytic functions, as previously described [28]. In that context, the recurrence of these loci in our results may align with the explanations proposed by those authors regarding the tendency of certain cellular maintenance pathways to appear within conserved genomic regions [25]. Likewise, the identification of SERAC1, SYNJ2, and SYTL3 which are involved in phospholipid remodeling, membrane turnover, and vesicular transport coincides with previous findings linking lipid metabolism and mitochondrial function to selection in indigenous breeds [9,10]. These shared loci may thus represent vestiges of early domestication events where energy balance and cell-membrane efficiency were advantageous under diverse environmental pressures.

In European populations, LIF, PPARGC1B, GRB10, and CSF1R were detected within recurrent ROH islands. These genes have been reported in local Mediterranean and Italian pigs, being associated with reproduction, metabolism, and immune performance [25,27,28]. LIF plays a crucial role in implantation and embryonic development; PPARGC1B regulates mitochondrial biogenesis and adipogenic differentiation; GRB10 modulates growth via insulin-like signaling; and CSF1R encodes the macrophage colony-stimulating factor receptor, essential for innate immune regulation [25,28]. The co-occurrence of these genes within ROH suggests selective maintenance of alleles related to fertility and metabolic efficiency, traits historically favored in European heritage breeds for prolificacy and high-quality fat deposition [14,27,28]. These findings support the idea that reproductive and metabolic traits have been major targets of selection in European pig lineages, aligning with the patterns observed in the Iberian-derived ESIB population, which exhibited the highest FROH (0.1543).

Conversely, the cosmopolitan group presented a distinct gene set ACP6, ANXA9, ARNT, BCL9, CDC42SE1, CTSS, GABPB2, HJV, ITGA10, MCL1, PDZK1, PI4KB, PIP5K1A, POLR3C, PSMD4, RFX5, SETDB1, SNX27, TXNIP, and VPS45 absent from all indigenous populations. Although only a limited number of homozygosity segments contained these loci shared with YBHP, several encode proteins linked to oxidative stress response (TXNIP), epigenetic regulation (SETDB1), apoptosis control (MCL1), and iron metabolism (HJV), processes typically associated with intensive metabolic selection and production efficiency in commercial pigs [10,11]. This functional contrast reflects the separation driven by modern breeding zootechnical purposes. Overall, the distribution of ROH-associated genes suggests that indigenous breeds tend to preserve a basic set of loci linked to robustness, developmental stability and metabolic balance, whereas cosmopolitan breeds exhibit selective and epigenetic genes linked to productivity.

For the YBHP, the presence of the shared indigenous genes (FGF5, BMP2K, PAQR3, RASGEF1B, ANTXR2) and the absence of cosmopolitan-exclusive loci further reinforce its genetic proximity to traditional European lineages and its potential as a reservoir of ancestral genetic diversity. However, due to the resolution limits of SNP-chip data, these associations should be interpreted as indicative rather than definitive or conclusive evidence, warranting validation through high-density sequencing and expression analyses. Additionally, sample size was not homogeneous among breeds, which may contribute to variability in ROH and FROH estimates; however, conservative filtering criteria were applied to reduce the impact of this factor.

## 5. Conclusions

YBHP shows ROH architecture dominated by medium-length segments and an intermediate FROH, indicating moderate inbreeding shaped by historical isolation and low-intensity selection. Its ROH overlap and candidate-gene profile align more closely with indigenous than cosmopolitan breeds, suggesting shared ancestry and/or convergent adaptation. The prioritized ROH hotspots and genes identified here offer concrete targets for fine-mapping and functional validation, and they support YBHP recognition as a unique genetic breed and a resource for conservation and sustainable use.

## Figures and Tables

**Table 1:** Populations used for this study.

Breed	Country	Continent	Code	Size	Type	References
Yucatán black hairless pig	Mexico	America	YBHP	141	Indigenous	Lara-Castillo et al. (2025) [14]
Ossabaw	Usa	America	USOS	6	Indigenous	Yang et al. (2017) [20]
Us yucatán mini pig	Usa	America	USYU	10	Indigenous	Yang et al. (2017) [20]
Kenya local	Kenia	Africa	KENYA	9	Indigenous	Yang et al. (2017) [20]
Bamaxiang	China	Asia	CNBX	20	Indigenous	Yang et al. (2017) [20]
Guangdongdahuabai	China	Asia	CNDH	20	Indigenous	Yang et al. (2017) [20]
Erhualian	China	Asia	CNEH	20	Indigenous	Yang et al. (2017) [20]
Lantang	China	Asia	CNLT	20	Indigenous	Yang et al. (2017) [20]
Meixan	China	Asia	CHMS	20	Indigenous	Yang et al. (2017) [20]
Angler sattleschwein	Germany	Europe	DEAS	10	Indigenous	Yang et al. (2017) [20]
Bunte bentheimer	Germany	Europe	DEBB	12	Indigenous	Yang et al. (2017) [20]
Chato murciano	Spain	Europe	ESCM	20	Indigenous	Yang et al. (2017) [20]
Iberian	Spain	Europe	ESIB	20	Indigenous	Yang et al. (2017) [20]
Mangalica	Hungary	Europe	HUMA	20	Indigenous	Yang et al. (2017) [20]
Mora romagnola	Italy	Europe	ITMR	9	Indigenous	Yang et al. (2017) [20]
Nero siciliano	Italy	Europe	ITNS	15	Indigenous	Yang et al. (2017) [20]
Pulawska	Poland	Europe	PLPS	15	Indigenous	Yang et al. (2017) [20]
Bisaro	Portugal	Europe	PTBI	14	Indigenous	Yang et al. (2017) [20]
Linderoth	Sweden	Europe	SELI	15	Indigenous	Yang et al. (2017) [20]
Duroc	Usa	Cosmopolitan	DUR	20	Cosmopolitan	Yang et al. (2017) [20]
Landrace	Denmark	Cosmopolitan	LDR	20	Cosmopolitan	Yang et al. (2017) [20]
Largewhite	Denmark	Cosmopolitan	LWT	20	Cosmopolitan	Yang et al. (2017) [20]
Pietrain	Netherlands	Cosmopolitan	PIT	20	Cosmopolitan	Yang et al. (2017) [20]
Hampshire	Uk	Cosmopolitan	HS	20	Cosmopolitan	Yang et al. (2017) [20]

**Table 2:** Summary of the number of runs of homozygosity (ROH) in different categories in each breed.

Breed	1–5 Mb	5–10 Mb	10–20 Mb	20–40 Mb	>40 Mb
YBHP	93	408	256	178	27
KENYA	25	122	52	10	0
USOS	9	85	58	7	1
USYU	21	149	81	23	0
CNBX	24	169	73	12	0
CNDH	46	215	115	37	2
CNEH	63	291	135	23	2
CNLT	50	297	162	29	5
CNMS	39	331	118	37	4
DEAS	22	165	78	21	0
DEBB	28	172	87	28	1
ESCM	42	248	129	19	2
ESIB	35	304	218	47	8
HUMA	38	236	137	33	1
ITMR	13	80	31	8	0
ITNS	11	126	62	14	2
SELI	13	105	65	18	3
PLPS	13	112	58	18	2
DUR	21	127	76	16	2
HS	28	189	138	29	3
LDR	27	168	141	34	5
LWT	18	154	122	27	4
PIET	22	160	86	35	5
PTBI	16	130	49	11	0

**Table 3:** Descriptive statistics for runs of homozygosity and inbreeding coefficients (FROH) within each breed.

Breed	Sample Size	Average Length (Mb)		Average Number		FROH	
		Mean ± SD	Range	Mean ± SD	Range	Mean ± SD	Range
YBHP	141	10.64 ± 7.46	2.93–79.67	19.19 ± 14.27	1–69	0.0906 ± 0.0800	0.0032–0.4656
KENYA	9	9.17 ± 4.98	2.79–37.34	23.22 ± 5.14	19–35	0.0945 ± 0.0213	0.0682–0.1411
USOS	6	10.48 ± 6.24	4.07–48.73	26.67 ± 9.50	12–38	0.1241 ± 0.0478	0.0507–0.1757
USYU	10	10.53 ± 6.23	3.76–39.67	27.40 ± 6.92	19–41	0.1280 ± 0.0315	0.0724–0.1780
CNBX	16	9.17 ± 4.37	3.90–25.95	17.38 ± 6.40	1–27	0.0707 ± 0.0291	0.0020–0.1205
CNDH	16	10.55 ± 6.71	3.32–67.99	25.94 ± 13.75	1–47	0.1215 ± 0.0723	0.0062–0.2535
CNEH	20	9.53 ± 5.61	3.14–58.71	25.70 ± 8.79	7–40	0.1087 ± 0.0409	0.0219–0.1785
CNLT	20	10.08 ± 6.01	2.97–50.27	27.15 ± 16.24	10–71	0.1214 ± 0.0974	0.0280–0.3970
CNMS	20	10.35 ± 5.96	3.34–45.58	29.60 ± 17.15	7–66	0.1360 ± 0.0966	0.0329–0.3732
DEAS	10	10.20 ± 6.10	3.38–38.95	28.60 ± 9.77	18–52	0.1294 ± 0.0472	0.0713–0.2446
DEBB	12	10.52 ± 7.10	3.49–70.37	26.33 ± 13.70	9–62	0.1229 ± 0.0869	0.0381–0.3643
ESCM	20	9.78 ± 5.38	3.47–43.10	22.00 ± 10.30	6–39	0.0955 ± 0.0523	0.0192–0.1699
ESIB	20	11.36 ± 7.67	3.33–74.27	30.60 ± 11.44	3–58	0.1543 ± 0.0676	0.0078–0.3197
HUMA	20	10.34 ± 6.29	3.76–73.23	22.25 ± 17.97	2–66	0.1021 ± 0.0982	0.0079–0.3874
ITMR	8	9.58 ± 5.41	4.08–30.36	16.50 ± 14.95	3–41	0.0701 ± 0.0756	0.0094–0.2009
ITNS	15	10.78 ± 7.30	3.33–52.52	14.33 ± 5.86	6–28	0.0686 ± 0.0365	0.0208–0.1392
SELI	15	11.28 ± 7.82	3.28–70.78	13.60 ± 11.19	2–43	0.0681 ± 0.0707	0.0067–0.2517
PLPS	15	10.93 ± 7.32	3.87–59.72	13.53 ± 12.83	2–47	0.0656 ± 0.0740	0.0063–0.2604
DUR	19	10.54 ± 6.55	3.28–45.94	12.74 ± 8.53	2–31	0.0596 ± 0.0476	0.0041–0.1795
HS	20	11.16 ± 7.34	3.75–78.11	19.35 ± 11.37	6–39	0.0958 ± 0.0645	0.0297–0.2343
LDR	20	11.90 ± 8.19	3.34–79.67	18.75 ± 12.75	4–47	0.0990 ± 0.0799	0.0139–0.2899
LWT	20	11.35 ± 6.67	3.32–54.60	16.25 ± 8.16	7–41	0.0819 ± 0.0486	0.0338–0.2414
PIET	20	11.76 ± 9.06	3.75–70.43	15.40 ± 10.59	2–48	0.0803 ± 0.0709	0.0047–0.2674
PTBI	14	9.40 ± 4.74	3.75–26.41	14.71 ± 7.65	3–32	0.0614 ± 0.0381	0.0106–0.1526

**Table 4:** Genes shared with the YBHP within ROH regions across worldwide pig populations.

Group	Chr	ROH	Snps	Genes	Enrichment Genes	Gene Names
**African**	8	4	95	14	12	BMP2K, FGF5, PAQR3, ENOPH1, GALNTL6, GPAT3, HNRNPD, SCD5, RASGEF1B, SEC31A, TMEM150C, ANTXR2
	14	2	52	1	0	
**American**	1	1	50	10	8	ANTXR2, BMP2K, FGF5, GTF2H5, IGF2R, PAQR3, RASGEF1B, SERAC1, SLC22A1, SNX9, SYNJ2, SYTL3
	8	2	58	7	4	
**Asian**	2	2	182	35	26	ABLIM3, ANTXR2, ARHGAP26, ARHGEF37, BMP2K, CAMK2A, CSF1R, CSNK1A1, DPYSL3, ENOPH1, FGF1, FGF5, GNPDA1, GRXCR2, HNRNPD, HTR4, KCTD16, LARS1, NDFIP1, NR3C1, PAQR3, PCDH12, PDE6A, PDGFRB, PPARGC1B, PPP2R2B, RASGEF1B, RNF14, SCD5, SEC31A, SH3RF2, SPINK5, SPRY4, TCERG1, TMEM150C
	8	3	88	13	9	
	9	1	54	2	0	
**Cosmopolitan**	4	3	54	29	24	ACP6, ANTXR2, ANXA9, AP1B1, ARNT, BCL9, BMP2K, CDC42SE1, CTSS, ENOPH1, EWSR1, FGF5, GABPB2, GJA5, GOLPH3L, HJV, HNRNPD, ITGA10, KREMEN1, LIF, MCL1, MN1, MTMR3, NEFH, NIPSNAP1, PAQR3, PDZK1, PEX11B, PI4KB, PIP5K1A, PITPNB, POLR3C, PSMD4, RASGEF1B, RFX5, SCD5, SEC31A, SETDB1, SNX27, TMEM150C, TXNIP, VPS45, ZNRF3
	8	3	88	13	7	
	14	15	54	13	12	
**European**	1	1	50	8	6	ABLIM3, ANTXR2, AP1B1, ARHGEF37, BMP2K, CAMK2A, CDX1, CSF1R, CSNK1A1, DEPDC5, DRG1, ENOPH1, EWSR1, EZR, FBXO38, FGF5, GAL3ST1, GPAT3, GRB10
	2	2	62	16	12	
	8	3	98	14	12	
	9	1	55	1	0	
	14	12	86	29	21	

## Data Availability

The data presented in this study are available on request from the corresponding author. Genotyping data are owned by the Asociación Mexicana de Criadores de Cerdos de Origen Iberico de Yucatán and can be shared after signing a Material Transfer Agreement.

## Supplementary Materials

The following supporting information can be downloaded at: https://www.mdpi.com/article/10.3390/vetsci13010054/s1, Table S1. Enriched Functional Categories in ROH-Associated Genes for Africa_RoH Populations. Table S2. Enriched Functional Categories in ROH-Associated Genes for Asian_RoH Populations. Table S3. Enriched Functional Categories in ROH-Associated Genes for American_RoH Populations. Table S4. Enriched Functional Categories in ROH-Associated Genes for European_RoH Populations. Table S5. Enriched Functional Categories in ROH-Associated Genes for Cosmopolitan_RoH Populations.
