# Supplementary material for: Exploring the Genetic Heritage of the Yucatán Black Hairless Pig: A Comparative Worldwide ROH Study
Source: Vet Sci. 2026 Jan 7;13(1):54. doi: 10.3390/vetsci13010054 (PMC12846694; doi:10.3390/vetsci13010054)
Supplement: Supplementary file 1 [file vetsci-13-00054-s001.zip › vetsci-4038118-supplementary.pdf]

Table 1. Enriched Functional Categories in ROH-Associated Genes for Africa_RoH Populations

Functional Category	GO Term(s)	GO Description(s)	Genes
Development	GO:0009653, GO:0032502, GO:0048856, GO:0050793	anatomical structure development ; anatomical structure morphogenesis ; developmental process ; regulation of developmental process	BMP2K; FGF5; PAQR3
Metabolism	GO:0009058, GO:0009893	biosynthetic process ; positive regulation of metabolic process	ENOPH1; FGF5; GALNTL6; GPAT3; HNRNPD; SCD5
Regulation	GO:0023051, GO:0023057, GO:0032879, GO:0040012, GO:0042752, GO:0048518, GO:0048519, GO:0048583, GO:0051239, GO:0065008, GO:0065009	negative regulation of biological process ; negative regulation of signaling ; positive regulation of biological process ; regulation of biological quality ; regulation of circadian rhythm ; regulation of localization ; regulation of locomotion ; regulation of molecular function ; regulation of multicellular organismal process ; regulation of response to stimulus ; regulation of signaling	BMP2K; FGF5; GPAT3; HNRNPD; PAQR3; RASGEF1B
Stimulus Response	GO:0009605, GO:0009719, GO:0042221	response to chemical ; response to endogenous stimulus ; response to external stimulus	FGF5; SEC31A; TMEM150C
Other	GO:0003008, GO:0007623, GO:0008283, GO:0009056, GO:0009628, GO:0016043, GO:0033036, GO:0040011, GO:0048511, GO:0048870, GO:0051234, GO:0051641, GO:0051674	catabolic process ; cell motility ; cell population proliferation ; cellular component organization ; cellular localization ; circadian rhythm ; establishment of localization ; localization of cell ; locomotion ; macromolecule localization ; response to abiotic stimulus ; rhythmic process ; system process	ANTXR2; BMP2K; FGF5; HNRNPD; PAQR3; SEC31A; TMEM150C

Table 2. Enriched Functional Categories in ROH-Associated Genes for American_RoH Populations

Functional Category	GO Term(s)	GO Description(s)	Genes
Cell cycle	GO:0022402	cell cycle process	SNX9
Development	GO:0009653, GO:0032502, GO:0048856, GO:0050793	anatomical structure development ; anatomical structure morphogenesis ; developmental process ; regulation of developmental process	BMP2K; FGF5; PAQR3; SYNJ2
Metabolism	GO:0009058, GO:0009893	biosynthetic process ; positive regulation of metabolic process	FGF5; GTF2H5; SNX9
Regulation	GO:0023051, GO:0023057, GO:0032879, GO:0040012, GO:0048518, GO:0048519, GO:0048583, GO:0051239, GO:0065008, GO:0065009	negative regulation of biological process ; negative regulation of signaling ; positive regulation of biological process ; regulation of biological quality ; regulation of localization ; regulation of locomotion ; regulation of molecular function ; regulation of multicellular organismal process ; regulation of response to stimulus ; regulation of signaling	BMP2K; FGF5; PAQR3; RASGEF1B; SLC22A1; SNX9
Stimulus Response	GO:0006950, GO:0009719, GO:0042221	response to chemical ; response to endogenous stimulus ; response to stress	FGF5; GTF2H5
Other	GO:0008283, GO:0009056, GO:0009628, GO:0016043, GO:0033036, GO:0040011, GO:0044085, GO:0044419, GO:0048870, GO:0051234, GO:0051641, GO:0051674	biological process involved in interspecies interaction between organisms ; catabolic process ; cell motility ; cell population proliferation ; cellular component biogenesis ; cellular component organization ; cellular localization ; establishment of localization ; localization of cell ; locomotion ; macromolecule localization ; response to abiotic stimulus	ANTXR2; BMP2K; FGF5; GTF2H5; IGF2R; PAQR3; SERAC1; SLC22A1; SNX9; SYNJ2; SYTL3

Table 3. Enriched Functional Categories in ROH-Associated Genes for Asian_RoH Populations

Functional Category	GO Term(s)	GO Description(s)	Genes

Development	GO:0009653, GO:0031128, GO:0032502, GO:0048589, GO:0048646, GO:0048856, GO:0050793, GO:0051093, GO:0051094	anatomical structure development ; anatomical structure formation involved in morphogenesis ; anatomical structure morphogenesis ; developmental growth ; developmental induction ; developmental process ; negative regulation of developmental process ; positive regulation of developmental process ; regulation of developmental process	BMP2K; CAMK2A; CSF1R; DPYSL3; FGF1; FGF5; GRXCR2; NDFIP1; NR3C1; PAQR3; PCDH12; PDE6A; PDGFRB; PPARGC1B; SPINK5; SPRY4
Immunity	GO:0002252, GO:0002376, GO:0002440, GO:0002520, GO:0002682, GO:0002683, GO:0002684, GO:0006955	immune effector process ; immune response ; immune system development ; immune system process ; negative regulation of immune system process ; positive regulation of immune system process ; production of molecular mediator of immune response ; regulation of immune system process	CSF1R; NDFIP1; PPARGC1B; SPINK5
Metabolism	GO:0009058, GO:0009893, GO:0042445	biosynthetic process; hormone metabolic process; positive regulation of metabolic process	ABLIM3; CAMK2A; CSF1R; CSNK1A1; ENOPH1; FGF1; FGF5; GNPDA1; HNRNPD; LARS1; NDFIP1; NR3C1; PDGFRB; PPARGC1B; RNF14; SCD5; SH3RF2; TCERG1
Regulation	GO:0023051, GO:0023057, GO:0030155, GO:0032879, GO:0040012, GO:0042752, GO:0048518, GO:0048519, GO:0048583, GO:0051239, GO:0051240, GO:0065008, GO:0065009	negative regulation of biological process ; negative regulation of signaling ; positive regulation of biological process ; positive regulation of multicellular organismal process ; regulation of biological quality ; regulation of cell adhesion ; regulation of circadian rhythm ; regulation of localization ; regulation of locomotion ; regulation of molecular function ; regulation of multicellular organismal process ; regulation of response to stimulus ; regulation of signaling	ABLIM3; ARHGAP26; ARHGEF37; BMP2K; CAMK2A; CSF1R; CSNK1A1; DPYSL3; FGF1; FGF5; HNRNPD; HTR4; KCTD16; LARS1; NDFIP1; NR3C1; PAQR3; PDGFRB; PPARGC1B; PPP2R2B; RASGEF1B; RNF14; SH3RF2; SPINK5; SPRY4
Reproduction	GO:0000003, GO:0003006, GO:0032504	developmental process involved in reproduction ; multicellular organism reproduction ; reproduction	NR3C1; PCDH12
Stimulus Response	GO:0006950, GO:0009605, GO:0009719, GO:0042221	response to chemical ; response to endogenous stimulus ; response to external stimulus ; response to stress	CAMK2A; CSF1R; DPYSL3; FGF1; FGF5; HTR4; LARS1; NDFIP1; NR3C1; PDGFRB; PPARGC1B; RNF14; SEC31A; SH3RF2; SPINK5; SPRY4; TMEM150C
Other	GO:0003008, GO:0007155, GO:0007610, GO:0007623, GO:0008283, GO:0009056, GO:0009607, GO:0009628, GO:0016043, GO:0019098, GO:0022414, GO:0033036, GO:0040007, GO:0040011, GO:0042330, GO:0044085, GO:0044419, GO:0045321, GO:0048511, GO:0048609, GO:0048870, GO:0050900, GO:0051234, GO:0051641, GO:0051674, GO:0051707	behavior ; biological process involved in interspecies interaction between organisms ; catabolic process ; cell adhesion ; cell motility ; cell population proliferation ; cellular component biogenesis ; cellular component organization ; cellular localization ; circadian rhythm ; establishment of localization ; growth ; leukocyte activation ; leukocyte migration ; localization of cell ; locomotion ; macromolecule localization ; multicellular organismal reproductive process ; reproductive behavior...	ABLIM3; ANTXR2; ARHGAP26; BMP2K; CAMK2A; CSF1R; CSNK1A1; DPYSL3; FGF1; FGF5; GNPDA1; GRXCR2; HNRNPD; KCTD16; LARS1; NDFIP1; NR3C1; PAQR3; PCDH12; PDE6A; PDGFRB; PPARGC1B; RNF14; SEC31A; SH3RF2; SPINK5; SPRY4; TMEM150C

Table 4. Enriched Functional Categories in ROH-Associated Genes for European_RoH Populations

Functional Category	GO Term(s)	GO Description(s)	Genes
Cell cycle	GO:0022402, GO:0051321	cell cycle process ; meiotic cell cycle	DRG1; EZR; HORMAD2; LIF; LIMK2; MN1; SNX9
Development	GO:0009653, GO:0032502, GO:0048589, GO:0048646, GO:0048856, GO:0050793, GO:0051093, GO:0051094	anatomical structure development ; anatomical structure formation involved in morphogenesis ; anatomical structure morphogenesis ; developmental growth ; developmental process ; negative regulation of developmental process ; positive regulation of developmental process ; regulation of developmental process	AP1B1; BMP2K; CAMK2A; CDX1; CSF1R; EZR; FBXO38; FGF5; GAL3ST1; KREMEN1; LIF; LIMK2; NEFH; OSBP2; PAQR3; PATZ1; PDE6A; PDGFRB; PPARGC1B; SH3TC2; SPINK5; SYNJ2; YWHAH; ZNRF3
Immunity	GO:0002252, GO:0002376, GO:0002440, GO:0002520, GO:0002682, GO:0002683, GO:0002684, GO:0006955	immune effector process ; immune response ; immune system development ; immune system process ; negative regulation of immune system process ; positive regulation of immune system process ; production of molecular	CSF1R; EZR; FBXO38; LIF; OSM; PATZ1; PPARGC1B; SPINK5

		mediator of immune response ; regulation of immune system process	
Metabolism	GO:0009058, GO:0009893	biosynthetic process ; positive regulation of metabolic process	ABLIM3; CAMK2A; CDX1; CSF1R; CSNK1A1; DEPDC5; DRG1; ENOPH1; EWSR1; EZR; FGF5; GAL3ST1; GPAT3; GRB10; GTF2H5; HNRNPD; LIF; LIMK2; MN1; OSM; PATZ1; PDGFRB; PISD; PPARGC1B; SNX9; TBC1D10A; YWHAH
Regulation	GO:0023051, GO:0023057, GO:0030155, GO:0032879, GO:0040008, GO:0040012, GO:0040013, GO:0042752, GO:0048518, GO:0048519, GO:0048583, GO:0051239, GO:0051240, GO:0065008, GO:0065009, GO:2000241	negative regulation of biological process ; negative regulation of locomotion ; negative regulation of signaling ; positive regulation of biological process ; positive regulation of multicellular organismal process ; regulation of biological quality ; regulation of cell adhesion ; regulation of circadian rhythm ; regulation of growth ; regulation of localization ; regulation of locomotion ; regulation of molecular function ; regulation of multicellular organismal process ; regulation of reproduc...	ABLIM3; ARHGEF37; BMP2K; CAMK2A; CDX1; CSF1R; CSNK1A1; DEPDC5; DRG1; EZR; FBXO38; FGF5; GPAT3; GRB10; HNRNPD; KREMEN1; LIF; LIMK2; MN1; NEFH; OSM; PAQR3; PATZ1; PDGFRB; PPARGC1B; RASGEF1B; SH3TC2; SNX9; SPINK5; TBC1D10A; YWHAH; ZNRF3
Reproduction	GO:0000003, GO:0003006, GO:0019953, GO:0032504	developmental process involved in reproduction ; multicellular organism reproduction ; reproduction ; sexual reproduction	GAL3ST1; HORMAD2; LIF; LIMK2; OSBP2; PATZ1
Stimulus Response	GO:0006950, GO:0009605, GO:0009719, GO:0042221	response to chemical ; response to endogenous stimulus ; response to external stimulus ; response to stress	CAMK2A; CSF1R; DEPDC5; EZR; FGF5; GRB10; GTF2H5; KREMEN1; MN1; MTMR3; NEFH; OSM; PDGFRB; PPARGC1B; SEC31A; SPINK5; TMEM150C; YWHAH
Other	GO:0003008, GO:0006914, GO:0007155, GO:0007623, GO:0008283, GO:0009056, GO:0009607, GO:0009628, GO:0016043, GO:0022414, GO:0033036, GO:0040007, GO:0040011, GO:0042330, GO:0044085, GO:0044419, GO:0044706, GO:0045321, GO:0048511, GO:0048609, GO:0048870, GO:0050900, GO:0051234, GO:0051641, GO:0051674, GO:0051707, GO:0098727	autophagy ; biological process involved in interspecies interaction between organisms ; catabolic process ; cell adhesion ; cell motility ; cell population proliferation ; cellular component biogenesis ; cellular component organization ; cellular localization ; circadian rhythm ; establishment of localization ; growth ; leukocyte activation ; leukocyte migration ; localization of cell ; locomotion ; macromolecule localization ; maintenance of cell number ; multi-multicellular organism process ; ...	ABLIM3; ANTXR2; AP1B1; BMP2K; CAMK2A; CSF1R; CSNK1A1; DEPDC5; DRG1; EZR; FBXO38; FGF5; GAL3ST1; GRB10; GTF2H5; HNRNPD; HORMAD2; KREMEN1; LIF; LIMK2; MN1; MTMR3; NEFH; NIPSNAP1; OSBP2; OSM; PAQR3; PATZ1; PDE6A; PDGFRB; PISD; PITPNB; PPARGC1B; SEC31A; SERAC1; SF3A1; SH3TC2; SNX9; SPINK5; SYNJ2; SYTL3; TBC1D10A; TCN2; TMEM150C; YWHAH; ZNRF3

Table 5. Enriched Functional Categories in ROH-Associated Genes for Cosmopolitan_RoH Populations

Functional Category	GO Term(s)	GO Description(s)	Genes
Cell cycle	GO:0022402, GO:0051321	cell cycle process ; meiotic cell cycle	LIF; MN1
Development	GO:0009653, GO:0032502, GO:0048589, GO:0048646, GO:0048856, GO:0050793, GO:0051093, GO:0051094	anatomical structure development ; anatomical structure formation involved in morphogenesis ; anatomical structure morphogenesis ; developmental growth ; developmental process ; negative regulation of developmental process ; positive regulation of developmental process ; regulation of developmental process	ACP6; AP1B1; ARNT; BCL9; BMP2K; CDC42SE1; FGF5; GJA5; KREMEN1; LIF; MCL1; NEFH; PAQR3; TXNIP; ZNRF3
Immunity	GO:0002376, GO:0002520, GO:0002682, GO:0002684, GO:0006955	immune response ; immune system development ; immune system process ; positive regulation of immune system process ; regulation of immune system process	ACP6; CTSS; LIF; POLR3C
Metabolism	GO:0009058, GO:0009893, GO:0042445	biosynthetic process ; hormone metabolic process ; positive regulation of metabolic process	ARNT; BCL9; ENOPH1; EWSR1; FGF5; GABPB2; HJV; HNRNPD; LIF; MN1; PI4KB; PIP5K1A; POLR3C; RFX5; SCD5; TXNIP
Regulation	GO:0023051, GO:0023057, GO:0030155, GO:0032879, GO:0040012, GO:0042752, GO:0048518, GO:0048519,	negative regulation of biological process ; negative regulation of signaling ; positive regulation of biological process ; positive regulation of multicellular organismal process ; regulation of	ARNT; BCL9; BMP2K; CDC42SE1; CTSS; FGF5; GABPB2; GJA5; GOLPH3L; HJV; HNRNPD; KREMEN1; LIF; MCL1; MN1; NEFH;

	GO:0048583, GO:0051239, GO:0051240, GO:0065008, GO:0065009, GO:0098900, GO:2000241	action potential ; regulation of biological quality ; regulation of cell adhesion ; regulation of circadian rhythm ; regulation of localization ; regulation of locomotion ; regulation of molecular function ; regulation of multicellular organismal process ; regulation of reproductive process ; regulation ...	PAQR3; PDZK1; PEX11B; PIP5K1A; POLR3C; RASGEF1B; RFX5; SETDB1; TXNIP; ZNRF3
Reproduction	GO:0000003, GO:0003006, GO:0032504	developmental process involved in reproduction ; multicellular organism reproduction ; reproduction	ARNT; LIF
Stimulus Response	GO:0006950, GO:0009605, GO:0009719, GO:0042221	response to chemical ; response to endogenous stimulus ; response to external stimulus ; response to stress	ARNT; CTSS; FGF5; GJA5; HJV; KREMEN1; MCL1; MN1; MTMR3; NEFH; PIP5K1A; POLR3C; SEC31A; TMEM150C; TXNIP
Other	GO:0003008, GO:0006914, GO:0007155, GO:0007623, GO:0008283, GO:0009056, GO:0009607, GO:0009628, GO:0016043, GO:0019882, GO:0022414, GO:0032259, GO:0033036, GO:0040007, GO:0040011, GO:0042330, GO:0044085, GO:0044419, GO:0044706, GO:0048511, GO:0048609, GO:0048870, GO:0051234, GO:0051641, GO:0051674, GO:0051707, GO:0098727	antigen processing and presentation ; autophagy ; biological process involved in interspecies interaction between organisms ; catabolic process ; cell adhesion ; cell motility ; cell population proliferation ; cellular component biogenesis ; cellular component organization ; cellular localization ; circadian rhythm ; establishment of localization ; growth ; localization of cell ; locomotion ; macromolecule localization ; maintenance of cell number ; methylation ; multi-multicellular organism pro...	ANTXR2; ANXA9; AP1B1; ARNT; BCL9; BMP2K; CDC42SE1; CTSS; FGF5; GJA5; GOLPH3L; HNRNPD; ITGA10; KREMEN1; LIF; MCL1; MN1; MTMR3; NEFH; NIPSNAP1; PAQR3; PDZK1; PEX11B; PI4KB; PIP5K1A; PITPNB; POLR3C; PSMD4; SEC31A; SETDB1; SNX27; TMEM150C; TXNIP; VPS45; ZNRF3
